# Evidence for Decreased Nucleolar PARP-1 as an Early Marker of Cognitive Impairment

**DOI:** 10.1155/2019/4383258

**Published:** 2019-11-19

**Authors:** Matthew Regier, Jiancong Liang, Alexander Choi, Kavita Verma, Jenny Libien, A. Iván Hernández

**Affiliations:** ^1^Department of Pathology, Downstate Medical Center, State University of New York, Brooklyn, NY, USA; ^2^Graduate Program in Neural and Behavioral Sciences, Downstate Medical Center, State University of New York, NY, USA; ^3^Department of Pathology, Microbiology and Immunology, Vanderbilt University Medical Center, Nashville, TN, USA; ^4^Department of Neurology, Downstate Medical Center, State University of New York, Brooklyn, NY, USA; ^5^Department of Neurology, Kings County Hospital, Brooklyn, NY, USA; ^6^The Robert F. Furchgott Center for Neural and Behavioral Science, Downstate Medical Center, State University of New York, Brooklyn, NY, USA

## Abstract

Poly(ADP-ribose) polymerase-1 (PARP-1) is a nuclear protein that regulates gene expression through poly(ADP)-ribosylation, resulting in the loosening of chromatin structure. PARP-1 enzymatic activity has been shown to be necessary for the expression of several genes required for memory formation and consolidation. Previously, we showed that nucleolar PARP-1 is significantly decreased in hippocampal pyramidal cells in Alzheimer's disease (AD). We proposed that the displacement of PARP-1 from the nucleolus results in downregulation of new rRNA expression and ribosome biogenesis, leading to cognitive impairment. To further investigate the relationship between nucleolar PARP-1 and memory impairment, we examined PARP-1 expression in the hippocampi of individuals with mild cognitive impairment (MCI) compared to control and AD cases. We used immunohistochemical techniques to examine the nucleolar distribution of PARP-1 in the Cornu Ammonis (CA region) of the hippocampus. PARP-1 positive cells were then scored for the presence or absence of PARP-1 in the nucleolus. We found a significant decrease of PARP-1 staining in the nucleolar compartment of hippocampal pyramidal cells in MCI compared with Control and AD. When the four CA (CA1-4) regions were considered separately, only the CA1 region showed significant differences in nucleolar PARP-1 with Control > AD > MCI cases. Categorization of nucleolar PARP-1 into “distinct” and “diffuse” groups suggest that most of the changes occur within the distinct group. In addition, measurements of the nucleolar diameter of nucleolar PARP-1 positive cells in CA2 and CA4 showed Control > MCI. Thus, MCI cases had a lower percentage of PARP-1 nucleolar positive cells in CA1 and smaller nucleolar diameters in CA2 and CA4, compared to Control. Our data suggest that disruption of nucleolar form and function is an early and important step in the progression of cognitive impairment.

## 1. Introduction

Alzheimer's Disease (AD) is a progressive neurodegenerative disorder that is characterized clinically by memory loss and cognitive impairment, and neuropathologically by extracellular aggregations of *β*-amyloid (A*β*) protein (neuritic plaques) and intracellular aggregations of hyper-phosphorylated tau protein (neurofibrillary tangles) [1, 2]. The amyloid cascade hypothesis, which posits that A*β* deposits lead to tau pathology and neurotoxicity, is the leading hypothesis of disease progression despite data that suggests a more complicated mechanism [2, 3]. For instance, although plaques and tangles are necessary for a neuropathologic diagnosis, the extent of these pathologies does not correlate well with cognitive decline early in disease progression [4]. To date, removal of A*β* or inhibition of its deposition has been the main target of drug development, but these drugs have not been able to improve cognitive performance over time [5]. A small number of drugs targeting other processes have been FDA approved for the treatment of AD, and the few approved exhibit limited therapeutic impact [6]. It is generally believed that many of the drugs tested in clinical trials fail because the therapy was started too late with respect to the progression of the disease [7–9]. Consequently, there is interest in identifying early (pre-clinical) markers of AD in order to re-test therapies that failed in later stages of the disease [3, 9]. This study was designed to explore the possibility of PARP-1 being one of those markers.

PARP-1 is a nuclear protein that utilizes NAD+ to synthesize poly(ADP-ribose), resulting in the modification of acceptor proteins [10]. First recognized for its role in DNA repair [11], it has since been shown that PARP-1 is a multifunctional protein that regulates diverse mechanisms ranging from heterochromatin structure and cell stress signaling to differential gene expression and ribosome biogenesis [12–15]. PARP-1 has been shown to be required for processes of synaptic plasticity, memory consolidation, reconsolidation and extinction [12, 16–18]. PARP-1 works as a chromatin remodeling enzyme allowing the expression of genes required for long-term synaptic plasticity and memory consolidation—for example, the immediate-early genes *c-fos* and *c-jun* and the nucleolar rRNA genes (rDNA) [12, 16, 17, 19–21].

We previously reported a significant reduction in the amount of nucleolar PARP-1 in hippocampal neurons in AD compared to age-matched controls and proposed that the loss of PARP-1 may be an early and consistent finding in AD [22]. The investigation of PARP-1 localization early in disease progression is important because of the role of PARP-1 in both cell survival and memory, two functions compromised in AD.

PARP-1 activation causes inhibition of DNA methyltransferase 1 (Dnmt1), preventing hypermethylation of DNA [23]. Previously, we proposed a model of AD in which the absence of nucleolar PARP-1 allows Dnmt1 to hypermethylate rDNA promoters [22]. The loss of nucleolar PARP-1 may explain the finding that rDNA genes are hypermethylated in mild cognitive impairment (MCI) and AD [24, 25]. Methylation is a key regulatory mechanism of gene transcription, and hypermethylation of rDNA promoters causes a reduction of ribosome biogenesis [26]. Transient disruption of ribosome biogenesis could cause memory impairments, as we have shown in mice [27], while persistent severe disruption can lead to cell death through insufficient protein synthesis capacity [28]. To investigate whether nucleolar PARP-1 could function as an early marker of cognitive impairment, we examined PARP-1 levels in the hippocampi of individuals with MCI compared to Control and AD cases.

## 2. Materials and Methods

### 2.1. Inclusion/Exclusion Criteria/Case Selection

Autopsy brain tissue was obtained from excess formalin-fixed, paraffin-embedded (FFPE) tissue from the Neuropathology services of SUNY Downstate Medical Center and Kings County Hospital for neuropathologically-confirmed AD cases and Controls. The Banner Sun Health Research Institute Brain and Body Donation Program of Sun City, Arizona provided FFPE brain tissue from individuals meeting the clinical criteria for amnestic MCI [29], neuropathologically confirmed AD, and Controls. Control tissues were from individuals without clinical history of dementia or cognitive dysfunction and had “Not” or “Low” levels of AD neuropathologic change [30]. See Table 1 for case summary data.

### 2.2. Staining

For PARP-1, immunohistochemical (IHC) staining of FFPE brain tissue was performed as previously described [22]. Briefly, the samples were de-paraffinized with xylene 3x 3 min, before being rehydrated in 100% anhydrous alcohol 2x 3 min followed by 95%, 70%, and 50% ethanol for 3 min each then rinsed in running tap water and distilled water. After rehydration, antigen retrieval was undertaken with 10 mM citrate buffer (pH 6.0) while being microwave irradiated for 15 min, followed by incubation with primary antibody (PARP-1 monoclonal antibody, 1:200; Cat # 1522G, AbD Serotec), then with biotinylated secondary antibody horse anti-mouse (1:200, Vector Laboratories), and developed using the ABC system (Vector Laboratories). H&E staining was performed as per standard protocol, briefly as follows: Sections were de-paraffinized with xylene 3x 3 min, before being rehydrated in 100% anhydrous alcohol 2x 3 min followed by 95%, 70%, and 50% ethanol for 3 min each. The samples were briefly rinsed in running tap water then distilled water. Staining was done with Richard-Allan Hematoxylin for 30s followed by a tap water/clarifier rinse for 30s, tap water rinse for 30s, bluing reagent for 1 min, another tap water rinse for 1 min, a rinse in 95% dehydrant alcohol followed by Richard-Allan Eosin-Y for 90s, 100% anhydrous alcohol for 3 min, and finally xylene for 3 min [31].

### 2.3. Light Microscopy

Sections were examined in a blinded fashion under light microscopy at 20x (N infinity/0.17/FN26.5 U1S-2) or with an oil immersion lens at 100x (UPlanFL N infinity/0.17/FN26.5 [100x/1.30]). Images were taken with an Olympus DP74 camera using the 100x lens on a Olympus BX51 light microscope using a non-overlapping scanning approach with at least one pyramidal neuron per photomicrograph. For each subject, approximately 10 non-overlapping images were captured for every subregion and all positively stained nucleoli in each image were measured and averaged in each CA subregion. This yielded one measurement per stain per subregion for each subject.

### 2.4. PARP-1 Intensity

**Figure 1:**
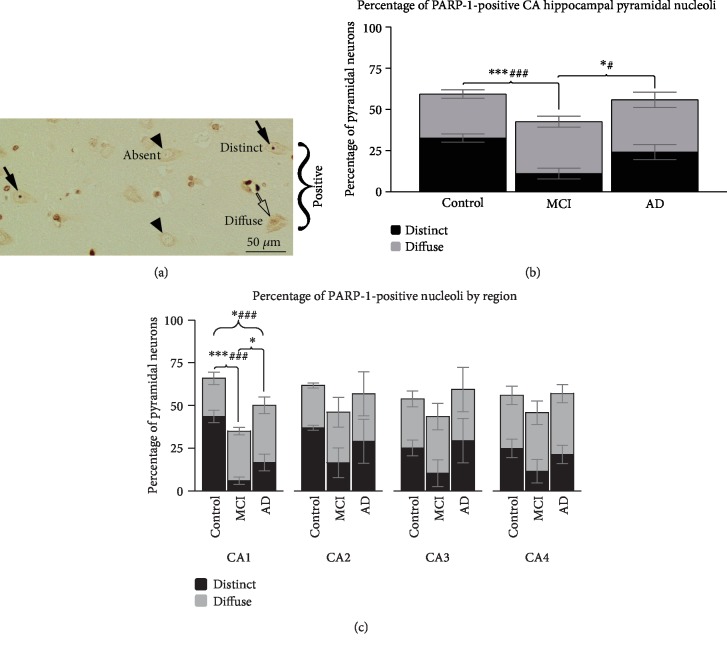
PARP-1 prevalence in CA pyramidal nucleoli is reduced in MCI. (a) Representative examples of pyramidal cells with distinct (closed arrows), diffuse (open arrows) or absent (arrowhead) PARP-1 stained nucleoli. (b) Percentage of PARP-1 positive nucleoli (distinct + diffuse) in the CA is lower in MCI compared to both Control and AD groups. The subset of PARP-1 nucleoli that had distinct (black bars) staining is also significantly lower in MCI compared to both Control and AD, whereas diffusely stained nucleoli (grey bars) show similar percentages. (c) Percentage of PARP-1 positive nucleoli in each of the four CA subregions. In CA1, PARP-1 positive nucleoli (distinct + diffuse) are smaller in MCI than AD, which are in turn smaller than Control. Other subregions do not show significant differences. When compared the subset of PARP-1 nucleoli that had distinct (black bars) staining, MCI and AD are both significantly lower compared to Control, whereas diffusely stained nucleoli (grey bars) show similar percentages. ^∗^ denotes significant differences with *p* < .05 for percentage of PARP-1 positive nucleoli. ^∗∗∗^ denotes significant differences with *p* < .001 for percentage of PARP-1 positive nucleoli. ^#^ denotes significant differences with *p* < .05 for percentage of distinct PARP-1 nucleoli subgroup. ^###^ denotes significant differences with *p* < .001 for percentage of distinct PARP-1 nucleoli subgroup.

PARP-1 positivity was determined by intensity above background nuclear stain and reviewed independently by another investigator. Background staining intensity was assessed and found not significantly different between groups. Positive nucleolar staining was divided into two categories, distinct and diffuse. The cutoff criterion for diffuse was any positive staining discerned within the nucleolar structure. The cutoff criterion for distinct was strong nucleolar staining in contrast to the nucleus (See Figure 1(a) and Supplementary [Supplementary-material supplementary-material-1]).

### 2.5. Nucleolar Diameter

Nucleolar diameter was computed on H&E or PARP-1 stained slides using DP Manager/Controller ruler on 100x images using the distance tool. For all discernible nucleoli, perpendicular short and long axes were measured by drawing straight lines and averaged to account for predominantly elliptical shape. In both H&E and PARP-1 stains diameter was measured to the outer extent of the nucleolus in order to include nucleolus-associated chromatin, which has been implicated in the spatial organization of active ribosomal genes and ribosome biosynthesis [32].

### 2.6. Statistics

#### 2.6.1. Quantification of Hippocampal Pyramidal Neurons with Distinct, Diffuse, or Absent

*PARP-1.* A multi-step process was used to statistically analyze PARP-1 nucleolar staining. First, an Analysis of Variance (ANOVA) was used to determine if diagnosis (Control, MCI, or AD) had an effect on PARP-1 staining (Distinct vs. Diffuse vs. Absent) across the whole CA (Figure 1(b)). Because a significant effect of diagnosis on PARP-1 staining was found for the whole CA, a Multivariate Analysis of Variance (MANOVA) test was used to analyze changes in the proportion of neurons with distinct, diffuse, or absent PARP-1 staining across individual CA subregions (CA1-CA4) (Figure 1(c)). For the CA as a whole, and CA subregions where a significant effect was found, pairwise comparisons were determined using post-hoc Tukey t-tests (Figure 1(c)).

#### 2.6.2. Quantification of Pyramidal Neuronal Nucleolar Size

**Figure 2:**
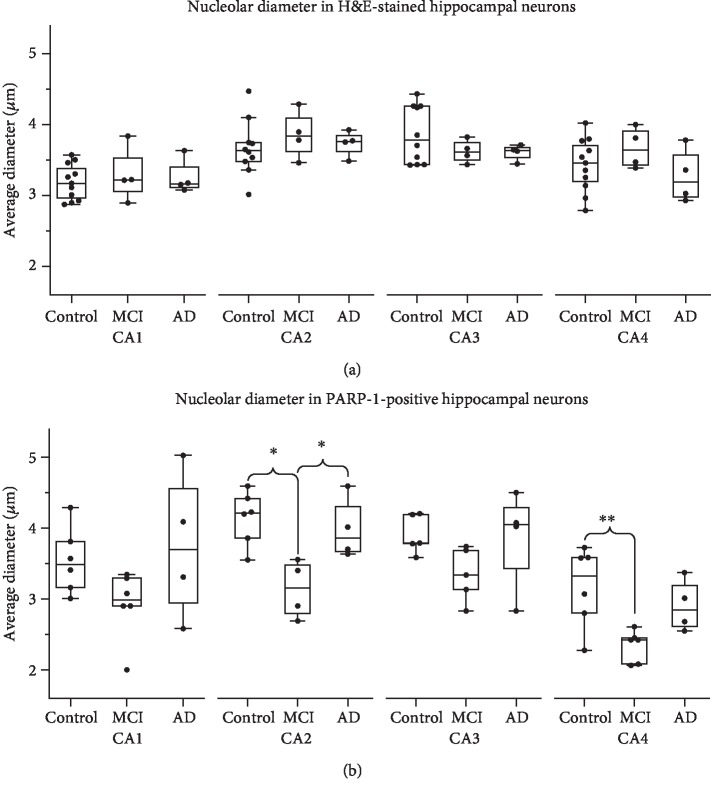
The average size of PARP-1 positive nucleoli is reduced in MCI in CA2 and CA4. (a) The average diameter of H&E stained nucleoli in CA1-CA4 shows no significant differences among Control, MCI and AD groups. (b) The average diameter of the subset of nucleoli positive for PARP-1 staining is smaller in CA2 neurons in MCI compared to Control and AD, while PARP-1 positive CA4 nucleoli are smaller in MCI than Control and differences between MCI and AD do not reach significance. Size of PARP-1 positive nucleoli in CA1 and CA3 are not different between groups. ^∗^ denotes *p* < .05. ^∗∗^ denotes *p* < .01.

A MANOVA was conducted to determine whether disease diagnosis (Control, MCI, or AD) had an effect on nucleolar size (measured as average nucleolar diameter) of H&E (Figure 2(a)) and PARP-1 (Figure 2(b)) staining in CA1-CA4 subregions. For subregions where significant differences were found, a post-hoc Tukey t-test was performed to determine pairwise significance. See Supplementary [Supplementary-material supplementary-material-1] and results section for all statistical data.

All statistical analysis was performed using IBM SPSS Version 21.0 and 24.0. Significance was assigned at *p* < .05 for ANOVA, MANOVA and Tukey tests. *P* < .05 is represented in figures by ^∗^, *p* < .01 is represented in figures by ^∗∗^^∗^, *p* < .01 is represented in figures by ^∗∗∗^.

## 3. Results

### 3.1. Nucleolar PARP-1 Is Less Prevalent in CA of MCI Cases

To characterize the percentage of nucleoli that are positive for PARP-1 across the Cornu Ammonis (CA) regions of the hippocampus (CA1-CA4), sections were stained with PARP-1 antibody and categorized as present or absent for PARP-1. Subsequently, PARP-1 positive nucleoli were further categorized as having “distinct” or “diffuse” staining (Figure 1(a), Supplementary [Supplementary-material supplementary-material-1]).

We found a reduction in the percentage of pyramidal cells with PARP-1 staining (distinct + diffuse) in the nucleoli of MCI cases compared to Control and AD cases across the CA (Figure 1(b); ANOVA: F_2,52_ = 7.819 *p* < .001. Tukey: MCI vs Control *p* < .001, MCI vs. AD *p* = .010). Control and AD cases did not have significantly different levels of PARP-1 positive nucleoli (Figure 1(b); Tukey: *p* = .475). Comparing PARP-1 positive nucleoli subcategories (distinct and diffuse staining) showed that the subset of PARP-1 positive nucleoli that had distinct staining was significantly lower in MCI cases compared to both Control and AD cases across the CA (Figure 1(b), Black bars; ANOVA: F_2,52_ = 11.972 *p* < .001. Tukey: MCI vs. Control *p* < .001, MCI vs. AD *p* = .011). As with PARP-1 positive nucleoli, distinct nucleoli were not significantly different in Control vs. AD cases (Figure 1(b), Black bars, Tukey: *p* = .091). There was no difference found in the percentage of nucleoli with diffuse staining in any diagnosis (ANOVA: F_2,52_ = .931 *p* = .4).

### 3.2. The Reduction of PARP-1 Positive Nucleoli Is Observed in CA1

We then analyzed the data for each of the four CA regions separately and found a significant difference in the percentage of PARP-1 positive nucleoli in CA1 (Figure 1(c), Left Panel; MANOVA: F_4,26_ = 5.81 *p* = .002), but not the other subregions (Figure 1(c). MANOVA: CA2: F_4,26_ = .848 *p* = .508, CA3: F_4,26_ = .744 *p* = .571, CA4: F_4,26_ = .877 *p* = .491). In CA1, we observed significant differences where MCI < AD < Control in percentage of PARP-1 positive nucleoli (Figure 1(c), Left Panel; MANOVA: F_2,13_ = 15.048 *p* < .001. Tukey: MCI vs. Control *p* < .001, MCI vs. AD *p* = .032, Control vs. AD *p* = .026). In comparing the distinct PARP-1 subgroup, we found a significantly lower percentage of nucleoli staining in MCI and AD cases compared to Control (Figure 1(c), Black Bars in Left Panel; MANOVA: F_2,13_ = 5.810 *p* = .002. Tukey: MCI vs. Control *p* < .001, AD vs. Control *p* < .001). Differences between distinct PARP-1 staining in MCI and AD cases show a trend, but did not reach significance (MCI vs. AD *p* = .058).

### 3.3. The Size of PARP-1-Positive Nucleoli Is Reduced in CA2 and CA4 of MCI Cases

We measured the diameter of the nucleolus of pyramidal cells in hippocampal regions CA1-CA4 to determine if nucleolar size was affected by MCI or AD. We found that the average size of CA neuronal nucleoli detected by H&E staining is not altered by disease state in any CA subregion (Figure 2(a); ANOVA: CA1: F_2,15_ = .103 *p* = .903, CA2: F_2,15_ = .396 *p* = .680; CA3: F_2,15_ = 1.258 *p* = .312; CA4: F_2,15_ = 1.257 *p* = .313). However, with PARP-1 immunostaining, we found a reduction in the nucleolar size of PARP-1 positive pyramidal neuronal nucleoli was altered by disease state in CA2 and CA4 but not CA1 or CA3 (Figure 2(b); ANOVA: CA1: F_2,13_ = 1.809, *p* = .214; CA2: F_2,13_ = 6.717, *p* = .014; CA3: F_2,13_ = 2.553, *p* = .127; CA4: F_2,13_ = 9.946, *p* = .004). A post-hoc pairwise analysis of PARP-1 positive nucleoli staining shows two subregions of the hippocampus with differences among groups. Whereas CA2 shows a reduction nucleolar size significantly different in MCI compared to the AD and Control groups, in CA4 MCI shows significant differences only with the control group. (Figure 2(b); Tukey: CA2: MCI vs. Control, *p* = .016; MCI vs. AD, *p* = .038; Control vs AD, *p* = .931; Tukey: CA4: MCI vs. Control, *p* = .003; MCI vs. AD, *p* = .082; Control vs AD, *p* = .191).

## 4. Discussion

### 4.1. PARP-1 as an Early Marker of Cognitive Impairment

The percentage of PARP-1 positive nucleoli in CA hippocampal pyramidal cells is lower in MCI cases compared to Control and AD. A closer look at PARP-1 positive nucleoli in the CA reveals that CA1 is the main subregion affected in the hippocampus. Dividing the nucleolar PARP-1 positive staining into “distinct” and “diffuse” shows the disappearance of PARP-1 to be primarily within the distinct subgroup. The finding of PARP-1 loss from the nucleolus in MCI cases dovetails with growing evidence of nucleolar function being compromised early in AD [24, 33]. In this study, CA1 is the most sensitive area of the hippocampus with the percentage of PARP-1 positive nucleoli lower in MCI cases than AD, and AD lower than Controls. The latter is consistent with our previous observation reporting a downregulation of PARP-1 positive staining in the hippocampal CA1 region in AD compared to controls [22]. These results are also consistent with previous observations in which CA1- subiculum was found to be involved early in spatiotemporal progression of AD atrophy and hypometabolism, and postulated to be particularly vulnerable due to high synaptic output and metabolic demand [34]. In this study, we did not find a decrease in the percentage of PARP-1 positive nucleoli in the CA4 region in AD, in contrast to our previously reported findings. The differences between studies could be due to the small number of cases, and further studies are necessary to determine PARP-1 presence in disease progression in the CA4.

A reduced size of the nucleolus in neurodegenerative diseases including AD and Parkinson's Disease has been reported and attributed to nucleolar stress [25, 35–39]. When the nucleolar diameter in hippocampal pyramidal cells was measured by H&E, we found no statistically significant differences. However, when the select group of nucleolar PARP-1 positive cells was measured, we found a reduced diameter of the nucleolus in the CA2 and CA4 region of MCI cases compared to Controls. Interestingly, the diameter of PARP-1 positive nucleoli is also lower in CA2 region of MCI cases compared to AD. Reduced nucleolar diameter found in PARP-1 positive nucleoli, but not all (H&E stained) nucleoli, supports the hypothesis that PARP-1 could be a marker of early cognitive impairment. The precise cause of PARP-1 positive nucleoli being more sensitive than total nucleoli to size changes in MCI is unclear. Future experiments will have to determine the mechanisms causing the differences in sensitivity.

### 4.2. The Role PARP-1 in Synaptic Plasticity and Learning and Memory

We and others have demonstrated that PARP-1 is important for synaptic plasticity and learning and memory [12, 16, 17, 19–21]. In *Aplysia,* we provided the first evidence that neuronal stimulation evokes PARP-1 dependent *de novo* rRNA synthesis [16]. We also found in mice, that PARP-1 dependent *de novo* rRNA synthesis is required for the maintenance of long-term potentiation, a physiological substrate of memory [21], and that learning induced *de novo* rRNA synthesis is required for memory consolidation in an active place avoidance hippocampal-dependent task [27]. Complementary to our studies, there is the work by Capitano and colleagues showing that inactivation of rDNA expression in hippocampus impairs memory [40]. Thus, these observations imply that nucleolar integrity and PARP-1 dependent biogenesis of ribosomes are required for long-term synaptic plasticity and memory consolidation. PARP-1 activity is certainly not sufficient for long-term synaptic plasticity and memory consolidation, but there is growing evidence to suggests it is one of the necessary proteins.

In AD, total protein synthesis, ribosomal number, and translation efficiency are reduced [41–43]. Furthermore, the nucleolus has been shown in some studies to have a smaller volume [38, 44], altered protein content [22, 33], and increased silencing of rDNA [24] indicating decreased nucleolar activity. Here, we show that the reduction of PARP-1 positive nucleolar staining in the CA1 region is significantly lower in MCI and AD cases compared to Control. Moreover, the nucleolar changes in MCI are more pronounced than AD cases, suggesting more severe alterations of PARP-1 dependent nucleolar function early in the progression of the disease. Whether significant neuronal loss occurs in MCI cases is unclear [45]. Even if MCI cases might present some cell loss [45] it cannot explain the drastic reduction of nucleolar PARP-1 positive staining. Given that PARP-1's exit from nucleoli is a sign of stress, we posit that the lower proportion of PARP-1 positive nucleoli in MCI compared to AD may be the result of an increase in stressed neurons in MCI that are eventually lost in AD progression. A similar theory has been proposed by Hetman's group to explain the finding that rDNA hypermethylation is more severe in MCI than AD [24]. Since PARP-1 has been shown to be important for rDNA expression, biogenesis of ribosomes, and epigenetic changes in nucleolar chromatin, it seems reasonable that rDNA hypermethylation and PARP-1 nucleolar absence in MCI and AD are linked.

In our previous work in AD, we hypothesized that nucleolar down-regulation of PARP-1 is an early event in AD leading to activation of Dnmt1, the silencing of rDNA, and as a consequence, memory deficits [22]. We also proposed that rather than just being a marker of the disease, the loss of nucleolar activity is an essential part of the cognitive deficits observed in AD. The data presented here is consistent with our hypothesis and provides evidence that decreased nucleolar PARP-1 is an early marker of cognitive impairment.

It is interesting that asymptomatic cases of AD (ASYMAD), in which individuals present no sign of cognitive impairment but have AD pathology, exhibit neuronal nucleolar hypertrophy [38, 44]. It has been suggested that this hypertrophy might be a compensatory mechanism that prevents cognitive impairment despite the AD neuropathology of neurofibrillary tangles and A*β* plaques. In our view, therapeutic approaches aimed at restoring nucleolar activity will be necessary to restore cognition in AD. Drugs that can induce nucleolar reactivation, mimicking ASYMAD nucleolar hypertrophy, may be useful for restoration of the protein synthesis machinery, which in turn will rescue cognition.

## 5. Conclusions

Here, we show that there is a reduction in the proportion of nucleoli that are PARP-1 positive in CA1 and the size of PARP-1 positive nucleoli is reduced during MCI in CA2 (compared to Control and AD) and CA4 (compared to Control only). The observation that different forms of nucleolar disruption (decrease in PARP-1 presence, or nucleolar size) are seen early in disease is an important finding that furthers the hypothesis that dysfunction of the nucleolus is a key step in the progression of AD. More work is required to continue to build on this hypothesis and develop a precise mechanism explaining how the nucleolus is disrupted, and how this disruption causes the deficits of AD. It is possible that nucleolar disruption impairs cognitive function directly by compromising the ability of cells to make memories and, later indirectly, by facilitating cell death. For the nucleolus to be a future target of AD therapeutic intervention, we must gain a better understanding of how its form and function change as cognitive impairment progresses in severity.

## Figures and Tables

**Table 1:** Demographic and diagnosis data for study cases.

Case #	Sex	Diagnosis	PARP-1 proportion	Nucleolar diameter	PARP-1 positive Nucleolar diameter	Age	Braak score
1	F	Control	✓	✓	✓	95	III
2	M	Control	✓	✓	✓	93	I
3	F	Control	✓	✓	✓	84	III
4	M	Control	✓	✓	✓	86	I
5	M	Control	✓		✓	71	II
6	M	Control	✓		✓	72	I
7	M	Control		✓		69	I
8	M	Control		✓		72	0
9	M	Control		✓		71	II
10	F	Control		✓		81	II
11	M	Control		✓		97	II
12	F	Control		✓		87	II
13	M	Control		✓		85	IV
14	M	MCI	✓	✓	✓	82	III
15	M	MCI	✓	✓	✓	97	III
16	F	MCI	✓	✓	✓	78	IV
17	F	MCI	✓		✓	90	IV
18	M	MCI	✓		✓	96	IV
19	F	MCI	✓		✓	93	IV
20	M	AD	✓	✓	✓	86	VI
21	M	AD	✓	✓	✓	90	V
22****	M	AD	✓	✓	✓	76	V
23	F	AD	✓		✓	88	V
24	M	AD		✓		89	IV-V

		Control	6	11	6	81.8	1.8
Summary data		MCI	6	3	6	89.3	3.7
		AD	4	4	4	85.8	5.1

## Data Availability

All data points used are made available as a supplementary file.

## Supplementary Materials

Supplementary Materials Supplementary Table 1 shows the collection of all statistical data presented (in Figures 1 and 2) in the manuscript and the Post-hoc (Tukey) analysis when significance was found in ANOVA. Supplementary Figure 1: examples of PARP-1 distinct, diffuse and absent staining.
