# Supplementary material for: Evidence for Decreased Nucleolar PARP-1 as an Early Marker of Cognitive Impairment
Source: Neural Plast. 2019 Nov 19;2019:4383258. doi: 10.1155/2019/4383258 (PMC6885846; doi:10.1155/2019/4383258)
Supplement: Supplementary Materials — Supplementary Table 1 shows the collection of all statistical data presented (in Figures 1 and 2) in the manuscript and the Post-hoc (Tukey) analysis when significance was found in ANOVA. Supplementary Figure 1: examples of PARP-1 distinct, diffuse and absent staining. [file 4383258.f1.pdf]

		Analysis of Variance		Post-hoc Tukey t-test, as apporpriate		
		Differences amongst all groups		Control Vs. MCI	Controls Vs. AD	MCI Vs. AD
		F statistic	P value	P value	P value	P value
**PARP-1 Presence (Fig 1)**						
Entire CA region	Present	7.819	< 0.001***	< 0.001***	0.475	0.010*
	Distinct	11.972	< 0.001***	< 0.001***	0.091	0.011*
	Diffuse	0.931	0.40	-	-	-
CA1	Present	15.048	< 0.001***	< 0.001***	0.026*	0.032*
	Distinct	5.810	0.002**	< 0.001***	< 0.001***	0.058
CA2	Present	0.848	0.508	-	-	-
	Distinct	-	-	-	-	-
CA3	Present	0.744	0.571	-	-	-
	Distinct	-	-	-	-	-
CA4	Present	0.877	0.491	-	-	-
	Distinct	-	-	-	-	-
**Nucleolar Diameter (Fig 2)**						
CA1	H&E Stained	0.103	0.903	-	-	-
	PARP-1 Stained	1.809	0.214	-	-	-
CA2	H&E Stained	0.396	0.680	-	-	-
	PARP-1 Stained	6.717	0.014*	0.016*	0.931	0.038*
CA3	H&E Stained	1.258	0.312	-	-	-
	PARP-1 Stained	2.553	0.127	-	-	-
CA4	H&E Stained	1.257	0.313	-	-	-
	PARP-1 Stained	9.946	0.004**	0.003**	0.191	0.082

Supplementary Table 1. Collection of all statistical data presented. Post-hoc Tukey analysis only if significance was found in ANOVA
